# Lay counsellors’ experiences of administering the AUDIT-C as a brief screening tool in a South African township

**DOI:** 10.1186/s12913-023-10230-2

**Published:** 2023-11-09

**Authors:** Lynne Goldschmidt, Buyisile Mncina, Malose Langa, Steven Rebello, Thokozile Budaza, Josephine Tshabalala, Tom Achoki

**Affiliations:** 1 Department of Psychology, University of the Witwatersrand, Johannesburg, South Africa; 2 Centre for the Study of Violence and Reconciliation, CSVR, Johannesburg, South Africa; 3 Private Practice, Johannesburg, South Africa; 4 ABInBev Foundation, New York, USA; 5 Africa Institute for Health Policy, Nairobi, Kenya

**Keywords:** AUDIT-C, Alcohol consumption, Lay counsellors, Alcohol treatment services

## Abstract

**Background:**

South Africa presents one of the riskiest patterns of alcohol consumption, with per capita consumption above the African regional average. Globally, there has been an increased focus on the potential of appointing lay counsellors to administer alcohol intervention strategies in resource-limited contexts. Given the increasing need for relevant and efficient intervention strategies in response to high-risk alcohol consumption, screening instruments such as the AUDIT-C have gained increased attention.

**Methods:**

This paper explores the experiences of 15 lay counsellors in response to the training received on how to administer the AUDIT-C instrument, as well as provide interventions such as brief advice or an appropriate referral, in the resource-limited South African township of Alexandra, Johannesburg. A focus group was facilitated for this purpose and, thereafter, a thematic content analysis was applied to identify the themes most central to the lay counsellors’ experiences.

**Results:**

The research findings suggest that the lay counsellors perceived the training to be adequate in preparing them for administrating the AUDIT-C and for providing any relevant interventions, and that their confidence in administering the instrument developed as the project progressed. However, recruitment and administration challenges were experienced in primary healthcare and community settings, and lay counsellors perceived home visits to be more appropriate with respect to issues related to confidentiality and stigmatisation.

**Conclusion:**

Overall, while lay counsellors feel that the training they received on the tool and the tool itself is useful for effectively implementing the AUDIT-C in low-resource communities, the availability and efficiency of alcohol treatment services in Alexandra Township need to be improved.

**Supplementary Information:**

The online version contains supplementary material available at 10.1186/s12913-023-10230-2.

## Background

South Africa presents one of the riskiest patterns of alcohol consumption, with per capita consumption above the African regional average [1]. Hazardous alcohol consumption is a major contributor to adverse health outcomes and risky behaviours [2]. These concerns, however, remain mostly neglected given the overburdened and under-resourced South African public healthcare system [2, 3]. This neglect highlights the need for intervention strategies that can be optimally applied within clinical settings without placing an additional burden on the limited resources. Globally, there has been an increased focus on the potential of appointing lay counsellors to administer alcohol intervention strategies in resource-limited contexts [4–7].

The Alcohol Use Disorders Identification Test (AUDIT) is a widely used instrument that was developed by the World Health Organization (WHO) for identifying risky or harmful alcohol consumption as well as alcohol dependence and abuse [8]. Given the need for effective procedures in low-resource contexts, abbreviated versions of the AUDIT have been developed that are shorter and more efficient [9–12]. The AUDIT-C is a shortened version of the original AUDIT and comprises the first three questions of the full scale. The AUDIT-C has been implemented in different Southern African settings, at times indicating greater sensitivity to risk than other instruments [13, 14]. Additionally, various studies have shown the AUDIT-C to have high reliability and validity [11, 15, 16]. The AUDIT-C has been used in various South African studies with varying results, depending on the level of risk and vulnerability of the population [13, 17–19].

Given the increasing need for relevant and efficient intervention strategies in response to high-risk alcohol consumption, screening instruments such as the AUDIT-C have gained increased attention [18, 20]. The current study aimed to contribute to the existing body of knowledge by exploring the experiences of lay counsellors pertaining to the training they had received on how to administer the AUDIT-C as a screening tool for identifying and modifying risky drinking behaviour as well as on providing brief advice or making a referral.

While the effectiveness of primary healthcare workers in administering the AUDIT-C has been explored, these studies were conducted in developed nations such as Australia and the United Kingdom [21, 22]. The results of this current study may offer some insight into perceptions of the effectiveness of the AUDIT-C in low-resource communities such as Alexandra Township. Furthermore, the findings provide an opportunity to, first, identify and understand any possible barriers that may be contributing to implementation gaps, and second, to facilitate the development of appropriate guidelines for the optimal use of the AUDIT-C in South African primary healthcare and community settings.

The study was conducted in Alexandra Township, Johannesburg, which was established in 1912. Alexandra Township has a rich history in relation to South Africa’s legacy of apartheid. The township was a prominent space for political organisation and resistance against the oppressive regime [23]. Despite its heritage, Alexandra has become characterised by various psychosocial factors, including poverty, high rates of crime and a lack of access to basic services [23, 24].

Langa [25] and Falkof [26] suggest that harmful alcohol consumption is prevalent in Alexandra, and that exposure and consumption start at an early age. Alexandra was therefore deemed an appropriate setting for the administration of the AUDIT-C.

## Methods

This study explored the lay counsellors’ experiences of the training received to administer the AUDIT-C in the resource-limited South African township of Alexandra, Johannesburg. The lay counsellors were recruited and employed by a non-profit organisation (NPO) that has been providing services in South Africa within the area of HIV/AIDS since 2002. The NPO was a key strategic partner of the Gauteng Provincial Department of Health and the Gauteng Provincial Department of Social Development and was therefore considered most suitable for recruiting lay counsellors familiar with the Alexandra community as well as for training them in the administration of the AUDIT-C and the additional provision of appropriate brief advice or referrals. The NPO formally contracted the lay counsellors to administer the AUDIT-C and they received a daily stipend.

A key recruitment criterion was that the lay counsellors were residents of the Alexandra Township to ensure that they were familiar with the context. At the onset of the initiative, six (6) of the lay counsellors had previous lay counselling experience while the majority were specifically trained to administer the AUDIT-C. Some of the lay counsellors had experience in HIV/AIDS intervention work and understood the intersection of alcohol with overall health and risky behaviour. The lay counsellors comprised twelve (12) females and three (3) males, their ages ranged between 22 and 49 years old. Three (3) of the lay counsellors administered the AUDIT-C by means of the HIV testing programme as well as the home-visiting programme only, whereas twelve (12) of the lay counsellors administered the AUDIT-C across the primary health care settings, as well as the HIV testing programme and the home-visiting programme.

Please see Table 1, which lists the respective project processes.

**Table 1:** AUDIT-C training and administration

Activity	Date
Training of lay counsellors	18 November 2020 – 20 November 2020
Pilot AUDIT-C Study	1 December 2020 – 15 December 2020
Audit-C Study	1 January 2021 – November 2021
Booster Training Session	27 January 2021
Additional Booster Training Session	17 March 2021
Training Feedback Session	23 November 2021

In response to the study’s exploratory requirements, a focus group approach was applied that was embedded in a qualitative framework. Qualitative research involves the production of descriptive data that is intended to provide an understanding of the meaning-making individuals assign to their experiences [27]. The focus group took place on one day, comprising of two sessions that had a cumulative duration of approximately 2 h. A semi-structured approach was applied to ensure the session remained focused on the experiences of the lay counsellors. Accordingly, the focus group was framed by two main questions, which were followed up with further probing questions in response to the experiences shared. First, the participants were asked to reflect on the training they had received on administering the AUDIT-C screening instrument and on providing brief advice and possible referrals. This was followed by more questioning to further explore whether the participants’ thought the initial three-day training session had been sufficient. Second, the participants were asked how the training had informed their experience of administrating the AUDIT-C. This was followed by questions to elicit feedback related to recruitment across the respective settings, as well as on how the screening process, the AUDIT-C score, and the relevant brief advice or referral intervention had been received. Further sets of questions explored the participants’ views on key aspects of the training and whether they had any additional recommendations to make concerning the effective implementation of the overall AUDIT-C administration process. The semi-structured interview schedule shows this in more detail [See Additional file 1].

The focus group was audio recorded and transcribed. Thematic analysis was applied as per the principles presented by Clarke and Braun [28]. This was followed by the processes of identification, coding, classification and analysis of themes [29, 30]. The first three authors immersed themselves in the data by reading and rereading the transcript independently of each other to assess whether they had identified similar or contrasting themes. This process served to enhance the trustworthiness of the data analysis. The potential themes were critically evaluated in relation to the research aim. As a result, the study presented themes that aligned with the research aim.

## Results

The following section serves to provide an overview of the themes that emerged from the group discussion facilitated with the lay counsellors with regard to the training received on administering the AUDIT-C and on providing the relevant intervention in response to the AUDIT-C score. Four central themes emerged, and these included (1) the role of training in the administration of the AUDIT-C, (2) different experiences across primary healthcare versus community settings, (3) AUDIT-C as a stepping stone to talking about alcohol use problems, and lastly (4) the perceived effectiveness of the AUDIT-C referral process on the levels of motivation of lay counsellors.

### The role of training in the administration of the AUDIT-C

The lay counsellors attended an initial three-day training workshop on the use of AUDIT-C that included the process of providing appropriate interventions such as giving brief advice or making a referral to local rehabilitation services. In the focus group, the lay counsellors made the following observations about the training.

*Yes, the training helped me to prepare for this work and ask people about their drinking and referral. – Lay counsellor 2*

*Yes, I must say the training helped me in understanding this AUDIT-C better. I was well prepared to use it. – Lay counsellor 8*

*The three-day training was sufficient, however, when new counsellors came by, because of the staff turnover challenges in the beginning of the project, the new counsellors relied on us to orient them into the process, and I feel like they may have needed their own training. – Lay counsellor 1*

In this study, two follow-up booster training sessions were scheduled over a period of two days, respectively, to assess the lay counsellors’ experiences in administering the AUDIT-C as well as to address any questions or difficulties they may have experienced. The two booster training sessions were also used to train newly recruited lay counsellors who replaced those that had dropped out after the first recruitment phase.

*I was now confident in using the scale that I found myself teaching new people on how to use the scale so that they do not make mistakes. – Lay counsellor 3*

Overall, there was mutual agreement that the instrument was easy to use once the lay counsellors had sufficient training in administering the AUDIT-C. Lay counsellors relied on the guidance and supervision of their coordinators to ensure the consistent quality of the data captured.

### Different experiences across primary healthcare versus community settings

The AUDIT-C has generally been used in clinical health settings [31]. Additionally, in this study, the AUDIT-C was administered in primary healthcare settings, an HIV testing programme, a home visiting programme and in informal community settings. The diverse settings presented a range of benefits and challenges.

The lay counsellors reported that the participants recruited at the primary healthcare settings were not open to talking about their drinking behaviours due to their concerns about being judged negatively by healthcare workers, as illustrated in the quotes below:

*Some people were here at the clinic due to other health problems, so it was not easy for them to talk about their drinking problem because they were worried about the nurses shouting at them for drinking while sick … Someone would just say - I don’t drink - while drinking. – Lay counsellor 7*

*Others would try and justify that it was a challenge to stop drinking and that they knew they should not be drinking … it was a challenge to get them to open up, but we encouraged them by assuring them that the information shared would remain confidential. – Lay counsellor 4*

In contrast, the lay counsellors reported that home visits allowed participants to speak freely about their experiences with drinking and the impact (if any) their alcohol consumption had on their lives or family relationships.

*Why don’t we get them in the comfort of their homes and screen them because they are more comfortable there? That is why you find out that the responses that you get in the homes are far better compared to the ones at the facilities … and I find that most of the ones from the facilities are not honest answers. – Lay counsellor 11*

According to the lay counsellors, the participants who were screened in their homes appeared to be more comfortable with discussing their drinking patterns and receiving brief interventions. Conversely, participants approached in informal community settings were not as accommodating and tended to rush the screening process.

*At times there were parts of the form that we skipped and would fill in later on like the date, and numbering because you could just tell that the participant was becoming irritable … sometimes you must balance … I have had someone leave because one person said it’s taking too long. – Lay counsellor 9*

Lay counsellors had to be flexible in community settings, especially when some participants did not complete the screening tool when they felt that the process took longer than anticipated. However, such conversations were also useful in psycho-educating the participants about their risky drinking behaviours.

### AUDIT-C as a stepping stone to talking about alcohol-use problems

Generally, the AUDIT-C is used as a quantitative instrument to collect basic facts about individual drinking patterns [9, 10, 12, 32]. In this study, the instrument also proved to be a stepping stone that allowed participants to share detailed stories with lay counsellors of their struggles with heavy alcohol consumption.

*What kept me going are the cases I used to come across, when people tell you about their stories, as to how they got into alcohol, and what kept them drinking. That then pushed me to say I can reach many people; I can help more people. – Lay counsellor 10*

*I must say I learnt a lot, and realise the diversity of the issue, the influences of alcohol and that people have different reasons why they drink alcohol. Others are unable to quit. Others see drinking alcohol as a way to detach themselves from society. There are a lot of reasons. So, it has taught me a lot of things in terms of how people think and the influence of alcohol. – Lay counsellor 3*

*I once spoke to a certain man who was around 64 years old if I remember well … and he told me that he started drinking when he was 17 years … so, from then he got to a point in his life where he is no longer working, his partner left him with the children and all that … so the only thing he did was drink. Because when he went to his friends also reminded him of his situation. When he wakes up, his mission is to get two (slang expression) … even when he works, his priority is to buy alcohol and then he will eat afterwards. His life is centred around getting detached from society, and the only way he can do that is by always drinking. – Lay counsellor 11*

*I also found that COVID-19 was a factor in high alcohol use as people started drinking more and more to stabilise the situation (cope). - Lay counsellor 14*

It is evident that AUDIT-C facilitated more detailed conversations beyond the three screening questions. In addition to answering questions, the participants provided detailed stories of when and how they started drinking alcohol and the problems this caused in their personal lives. Lay counsellors asserted that they needed to be non-judgmental in their approach as a way of encouraging help-seeking behaviour amongst those individuals assessed as moderate- and high-risk drinkers.

*There was this woman who said she wanted to approach us but withdrew when she thought about how much she drinks. So, we had to teach her that do not call them drunkards when you approach them. Try to talk to those people nicely … don’t give up on people. One’s approach makes it hard for people to seek help. The way you approach them would make it easy for them to see that there is help out there, and there is somebody you can rely on, that if I were with this person, I would get help. – Lay counsellor 4*

*One participant mentioned to me that they are afraid to be seen around the referral centre as anyone who knows them will automatically assume that they are struggling with alcohol abuse and call them a drunkard. – Lay counsellor 10*

*Someone would say no not going there [rehabilitation centre] because many are going to laugh at me. People don’t want to be seen at that place. - Lay counsellor 7*

*When I referred a participant, they asked if they would be assisted by me because of how they felt when we were talking (rapport) and when I mentioned that someone else would assist, they were suddenly hesitant – Lay counsellor – 13*

While some individuals were evidently keen to seek help, the stigma associated with heavy drinking appeared to be a barrier. Heavy drinkers reported being called derogatory names such as ‘drunkard’ by members of the community and intimately by their own families and friends. Lay counsellors asserted that non-judgmental conversations using the AUDIT-C may facilitate such interventions and encourage people to seek help. The lay counsellors described how some participants were able to seek help and stop drinking.

*The lady that I was seeing is no longer drinking, now she is selling popcorn, so it’s like those things. Yes, it was impactful in a sense that, sometimes even when the champions are just walking. She was staying on the street, drinking alcohol, and smoking nyaope. I saw her, she is working, she is gaining weight. Now she is clean, she is no more drinking too much alcohol. – Lay counsellor 11*

*I stay in a flat. So, I was happy in one instance when a councillor [community leader] said we know you are the only person who can help that person [a lady who was known to drink heavily]. So that thing [SBI work] afforded us a level of recognition. So, if you can just train us broadly, so we can refer less and deal with the problem. – Lay counsellor 9*

*Many participants that have screened that need referral have tried to go and get a referral, but sometimes they were turned back by the stakeholders and some of them had really changed their lives because some of them had successful stories. – Lay counsellor 6*

Some participants were able to stop drinking after brief interventions as demonstrated in the excerpts above. The lay counsellors explained that their confidence increased and that they aspired to develop their skills to directly assist participants rather than simply refer them to rehabilitation services.

Despite these hopeful engagements, lay counsellors also reported that some participants appeared to be proud of their heavy drinking, with comments such as ‘everyone drinks here in Alex’.

*I had a lot of experiences where a person would just be boastful about how much they drink. When you tell him how high his score is, he will be proud. They become boastful about that. – Lay counsellor 5*

*Some people were proud that they drink so many beers on weekends. – Lay counsellor 3*

*Another factor to high alcohol consumption is the lack of self-awareness, as they drink because that is all they know and they grew up around alcohol and as such, they did not see any problem with their risky alcohol use. - Lay counsellor 15*

These boastful heavy drinking narratives were shared mainly by young black men who interpreted these risky behaviours as part of constructing their township masculinities [33]. Drinking ‘many beers on weekends’ is a symbol of successful masculinity in the socio-economic context of Alexandra. Young men often compete amongst themselves about who has managed to drink many beers. These excerpts represent some of the contextual, social, and systemic dynamics that need to be taken into consideration in future intervention strategies, especially when engaging young men about their risky drinking behaviours.

### Perceived effectiveness of the AUDIT-C referral process and its impact on the lay counsellors motivation

Lay counsellors were expected to refer participants who were classified as engaging in harmful drinking patterns to rehabilitation centres. At the outset of the study, three rehabilitation facilities were identified for participants who needed help. However, concerns were raised about a lack of feedback after referrals had been made.

*I wish we were getting feedback from the organisation we work with. I wish we knew how many people we referred actually went, are we on the right track or not. – Lay counsellor 4*

*Lack of motivation is the same thing – they say that you try to help a person and you make referrals, but you never know whether they go or not. – Lay counsellor 12*

*We have this long list of people who have been referred, but we don’t know if they actually go. And, if they go some of them are like, ‘They turned me away’. Another one would say, ‘I went, and they said they would call me. I am still waiting for a call’. The communication is just bad. – Lay counsellor 9*

The findings suggest that the motivation of lay counsellors is significantly influenced by the extent to which they feel that their efforts in referring help-seeking individuals are appreciated [34, 35], as demonstrated in the following excerpts.

*What I noticed is that all the people went for referrals. They went to SANCA, and also came back to me. Overall, I would say people are happy with the project. Especially when you give them better options and that you care, and you want to help them where you can. I feel that other than those people who were scornful, a lot of people appreciated what we were offering them. I feel it was more of a service delivery as a community to enable people to access help. But apart from that, I feel the programme was good. – Lay counsellor 3*

*Yes, two of the people I referred said to me that they went and are now clean and not drinking. – Lay counsellor 5.*

Other lay counsellors expressed concerns about the capacity of these referral sources to provide rehabilitation services.

*I once referred a participant to the clinic for assistance with their drinking and they came back and told me that they waited to be attended to the entire day, and were eventually turned back without getting the assistance. – Lay counsellor 3*

The findings suggest that some of the participants who had accepted referrals to rehabilitation centres successfully managed to deal with their drinking problems. However, while these individual success stories are to be applauded, rehabilitation services are not generally available and accessible in townships such as Alexandra [36].

## Discussion

The overall aim of the current study was to explore lay counsellors’ experiences with the training received, and how the training informed their experience of administering the AUDIT-C. The findings provide an understanding of the lay counsellors’ experience of the training they received, as well as their experiences of the AUDIT-C administration process across various settings. The central themes demonstrate that the lay counsellors had experienced the training as sufficient in relation to the immediate requirements of administering the AUDIT-C. This aligns with the findings of previous studies that had also demonstrated lay counsellors’ efficiency in administering alcohol screening instruments [5–7]. In this study, however, there were various contextual and situational dynamics that appeared to have influenced lay counsellors’ experiences in administering the AUDIT-C as compared to international studies in better resourced contexts [9]. Lay counsellors experiences in the current study are therefore likely to be beneficial in resolving any possible implementation gaps as well as inspiring further training components in resource-limited contexts.

Research suggests that residents in South African townships are at risk of participating in high levels of alcohol consumption [37, 38]. This is attributed to various psychosocial factors linked to poverty, such as food insecurity and unemployment. Additionally, research indicates that high levels of alcohol consumption are often linked to binge drinking [39–41]. This finding is in turn associated with psychosocial concerns related to violence, including sexual violence, as well as to risky sexual behaviour and HIV prevalence [37, 40, 42, 43]. These local findings align with international research [1] asserting that lower socio-economic segments of communities are at greater risk of ‘harm per litre’ of alcohol.

Screening tools such as the AUDIT-C have been applied across various contexts to ascertain its potential to reduce harmful alcohol consumption. In the United Kingdom, Dhital et al. [21] recommended that community pharmacists, nurses and doctors should be given regular training on the AUDIT-C to identify harmful alcohol consumption and refer those who need help to specialised rehabilitation services. This recommendation is confronted with various barriers in the South African context, given that the South African public healthcare system is overburdened with various other health concerns [2, 3]. These include HIV/AIDS, hypertension, injuries, and other common ailments. Additionally, research specific to the South African context indicates a need to focus on the sensitive provision of care amongst healthcare providers, which appears to be influenced by difficult working conditions. Schneider et al. [44] found that the nurses interviewed in their study had a limited understanding of alcohol consumption among patients who attended healthcare facilities in the Western Cape, South Africa. As a result, they had negative attitudes towards patients with HIV who were also drinking heavily. These findings align with the lay counsellors’ reports that participants worried about being reprimanded by clinic staff about consuming alcohol whilst on treatment.

Schneider et al. [44] concluded that healthcare workers need special training to effectively diagnose alcohol-use disorders and provide brief interventions for treating alcoholism. This need is confounded given that stigma and negative beliefs about alcohol treatment are prevalent in low-income communities in Cape Town, South Africa [45]. These factors may impact negatively on people’s motivation to seek help. The aforementioned research aligns with the findings of this research initiative situated in Alexandra, South Africa, where participants expressed their fears about being reprimanded by nurses for consuming alcohol. These expressed fears and concerns provide an understanding of why lay counsellors experienced participants as being more forthcoming about their alcohol consumption during home visits in comparison to participants screened in public health care settings.

In contrast with the participants’ fears of being reprimanded by nursing staff in the public health care settings, the reflections by the lay counsellors suggest that the participants’ experiences with the lay counsellors and the AUDIT-C facilitated a more in-depth engagement pertaining to participants’ lived experiences and how the participants made meaning of their difficulties with excessive alcohol consumption. It may therefore be suggested that the lay counsellors’ detachment from formal settings such as public health care clinics and rehabilitation services may have made them more accessible to participants in need of intervention.

The research findings, therefore, demonstrate the potential of lay counsellors in facilitating alcohol intervention services. Whilst the appointment of lay counsellors may initially have been proposed to relieve the already burdened health care system, their availability has also offered a service that is disconnected from services associated with stigma. The findings also highlight the role of destigmatising services and how this may encourage help-seeking behaviour.

Research has determined that, although these interventions are brief, they are found to be effective in reducing the level of drinking by 60% [46]. Research findings by Bradley et al. [9] indicate that the AUDIT-C can be utilised easily by frontline workers to assess risky drinking behaviours amongst individuals who visit healthcare centres provided they have been trained well. Similarly, Dhital et al. [21] and Tam et al. [22] asserted that lay counsellors must be trained regularly on using the AUDIT-C with the general population. All lay counsellors participating in this study indicated that the initial training conducted over a three-day period was sufficient in preparing them to administer the AUDIT-C instrument.

Taking into consideration the central role of lay counsellors in the intervention process, it is key to assess factors that may influence their levels of motivation in administering the AUDIT-C. The narratives shared by the lay counsellors suggest that having clarity on the referral outcome was key to their own buy-in on the intervention process. Consequently, lay counsellors were at times discouraged when there was a lack of transparency in communication, as well as poor service provision and follow-through by rehabilitation services. This study, therefore, highlights the need to review the accessibility and relevance of alcohol treatment centres and services in low-resourced contexts such as the Alexandra Township in South Africa.

## Strengths and limitations of the study

Only a few studies of this nature have been conducted in lower-income contexts characterized by high alcohol consumption. This findings of this study present new data useful for the efficient implementation of interventions that focus on alleviating treatment gaps in low-resourced contexts. The findings of this study have been shared with the Department of Health, which, in collaboration with the City of Johannesburg, plans to recruit and train lay counsellors on the use of AUDIT-C instrument to provide brief interventions in diverse communities.

A further strength of this study was the participatory approach taken with the lay counsellors, which involved regular reflective sessions to discuss the research process and its related challenges. These feedback sessions all served as an opportunity to learn and enhance the quality of the data collection process.

A possible limitation of this study is that even though a participatory approach was applied, the focus group component did not include a participant transcript review process. On the other hand, whilst participant transcript reviews offer potential benefits, they also present possible drawbacks [47].

The training and research were carried out during different waves of the COVID-19 pandemic. South Africa implemented various restrictions on the sale of alcohol across the sequential waves of the pandemic [48, 49] and this would likely have had an impact on the lay counsellors’ experience of both the training as well as their administration of the AUDIT-C. The research findings should therefore be reviewed in relation to the COVID-19 pandemic and its associated restrictions. It is important to note that these restrictions may also have influenced the drinking patterns of the participants with whom the lay counsellors interacted.

A further limitation is that the findings reflect the experiences of a small sample of lay counsellors, and the findings should therefore not be generalised to cover the experiences of all lay counsellors providing services in low-sourced South African settings.

Despite these limitations, the key findings of this study point to the benefits of training lay counsellors on implementing AUDIT-C as a screening device.

## Conclusions

South Africa presents an alarming trend of high-risk alcohol consumption, which is in turn associated with various psychosocial problems. Though the need for intervention is recognised, responding effectively presents a challenge to South Africa’s under-resourced public healthcare system [2]. Lay counsellors are therefore central to extending healthcare services and interventions in under-resourced contexts in South Africa [34]. The exploration of lay counsellors’ experiences in administering the AUDIT-C in Alexandra Township, South Africa, therefore provides important learnings for future intervention implementation.

The research findings suggest that while the lay counsellors perceived their training to be appropriate, the project experienced various challenges administering the AUDIT-C instrument effectively in different settings. Challenges with conducting the screening were particularly evident in primary healthcare and community settings. In contrast, lay counsellors perceived home visits to be a more favourable setting for conducting the AUDIT-C. This perception was attributed to factors related to confidentiality and the stigmatisation of heavy drinkers. Despite these concerns, the lay counsellors considered the AUDIT-C to be helpful for many of the participants because they used the screening to reflect on their drinking patterns.

The study demonstrates the potential of lay counsellors in facilitating alcohol intervention services. This is of importance in low resourced contexts, as prevalent in South Africa, given the potential to relieve the already burdened health care system.

### Supplementary Information

**Additional file 1.** Semi—structured Group Interview Schedule.

## Data Availability

The data that support the findings of this study are available from the ABInBev Foundation, but restrictions apply to the availability of the data. Data are however available from the authors upon reasonable request and with permission of the ABInBev Foundation.

## Abbreviations

AUDIT: Alcohol Use Disorders Identification Test
AUDIT-C: Alcohol Use Disorders Identification Test – Consumption
NPO: Non-Profit Organisation
SBI: Screening and Brief Advice
WHO: World Health Organization
