# Supplementary material for: Lay counsellors’ experiences of administering the AUDIT-C as a brief screening tool in a South African township
Source: BMC Health Serv Res. 2023 Nov 9;23:1227. doi: 10.1186/s12913-023-10230-2 (PMC10633970; doi:10.1186/s12913-023-10230-2)
Supplement: Supplementary file 1 — Additional file 1. Semi—structured Group Interview Schedule. [file 12913_2023_10230_MOESM1_ESM.pdf]

Semi—structured Group Interview Schedule

Introduction

The purpose of this session is to reflect on the training received, and how the training may have informed the overall experience of the project.

The role of training

1) Explore the role of training in administering the AUDIT-C screening instrument and in providing brief advice and possible referrals
    a) Explore the role of training and its effectiveness
    b) Expectations post-training compared to the administration experience
    c) How did you experience the 3-day training session?
    d) How did the 3-day training session inform your approach to implementing the SBI?
    e) Throughout the project, we had several check-ins/ booster sessions to support your process and maintain the integrity of the data collected. What was your experience of these sessions?

Different experiences across clinic / community settings

2) Explore the experiences across the clinic / community settings
    a) Reception by community members across different settings
    b) Experience working with other key community stakeholders, clinics, counselling centers, etc.
    c) As the study progressed, what were some of the challenges experienced in the community?

AUDIT-C as a steppingstone to talking about alcohol use problems

3) Alcohol use and abuse in South Africa is a well-known challenge that has a profound impact on families, communities, and the broader society. For this segment, it would be important to speak about the self-awareness participants had regarding alcohol use patterns in Alexandra.
    a) The project was about screening and brief intervention, how did participants understand the impact of alcohol use patterns on their lives, the lives of others as well as their livelihoods?
    b) Are people aware of the impact harmful alcohol use has on their lives?
    c) Were participants willing or resistant to receiving further interventions that were recommended in the project?

Perceived effectiveness of the AUDIT-C referral process/Motivation and referral

4) To explore the personal reflections of the implementers on the effectiveness of this project

Closing

Thank you so much for your honest responses to this and we wish you well for the remainder of the year.
